# Mild Prolongation of Prothrombin Time Does Not Affect the Safety and Prognosis of Transjugular Intrahepatic Portal Shunt: Based on Real-World Data

**DOI:** 10.5152/tjg.2023.22410

**Published:** 2023-08-01

**Authors:** Yaowei Bai, Jiacheng Liu, Yang Chen, Chongtu Yang, Yingliang Wang, Chaoyang Wang, Shuguang Ju, Chen Zhou, Songjiang Huang, Tongqiang Li, Wei Yao, Jinghong Yao, Bin Xiong

**Affiliations:** 1 Department of Radiology, Union Hospital, Tongji Medical College, Huazhong University of Science and Technology, Wuhan, China; 2 Hubei Province Key Laboratory of Molecular Imaging, Wuhan, China; 3 Department of Infectious Diseases, Union Hospital, Tongji Medical College, Huazhong University of Science and Technology, Wuhan, China

**Keywords:** Prothrombin time, transjugular intrahepatic portosystemic shunt, liver cirrhosis

## Abstract

**Background/Aims:**

The aim was to investigate the safety and prognosis of transjugular intrahepatic portal shunt in patients with mildly prolonged prothrombin time.

**Materials and Methods:**

Two hundred fifty-three patients with portal hypertension who received transjugular intrahepatic portal shunt from November 2015 to May 2021 in Wuhan Union Hospital were retrospectively selected. According to the preoperative prothrombin time, they were divided into 2 groups: 126 patients in the non-clinical significance group (prothrombin time prolongation <3 seconds) and 127 patients in the clinical significance group (3 seconds ≤ prothrombin time prolongation <6 seconds). A line chart of postoperative liver and kidney function was drawn, and Kaplan–Meier curve was used to analyze and compare the prognosis of the 2 groups.

**Results:**

Transjugular intrahepatic portal shunt was successfully performed in all patients; the technical success rate was 100%, and no puncture-related complications occurred during perioperative period. The mean preoperative prothrombin time was 14.9 ± 0.7 seconds in the non-clinical significance group and 17.2 ± 0.8 seconds in the clinical significance group. During follow-up, 1-year stent dysfunction rates in the non-clinical significance group and clinical significance group were 3.5% and 6.9%, respectively, with no statistically significant difference (hazard ratio = 0.77, 95% CI = 0.30-1.93, log-rank *P* = .575). In addition, there were no significant differences in the cumulative survival rate (log rank *P* = .255), rebleeding rate (log-rank *P* = .392), and incidence of hepatic encephalopathy (log-rank *P* = .404) between the 2 groups. Subgroup analysis of the clinical significance group showed no significant difference in safety and prognosis between the 2 subgroups.

**Conclusion:**

Transjugular intrahepatic portal shunt is safe for portal hypertension patients with prothrombin time prolongation <6 seconds. There was no significant difference in prognosis between the non-clinical significance group and the clinical significance group.

**Main Points**

- A strength of this research is that this study had a large sample size and a high follow-up rate.
- This study investigated the safety of transjugular intrahepatic portosystemic shunt in patients with coagulopathy, an issue not explained in the guidelines.
- There is no need to correct the patient’s clotting function before transjugular intrahepatic portal shunt in order to avoid delay of therapy.

## INTRODUCTION

Cirrhosis is a common disease of the digestive system. It is necrosis and fibrosis of the liver caused by different etiologies. Histologically, it is characterized by diffuse nodular regeneration, dense fibrous septum around, and inordinate vascular structure of the liver.^1^ The decline of the liver function will lead to the reduction of coagulation factor synthesis and the decrease in the platelet count,^2^ and patients with portal hypertension cirrhosis are often accompanied by different degrees of coagulation dysfunction.^3^ At the same time, gastrointestinal bleeding is a common symptom in patients with portal hypertension. Once gastrointestinal bleeding occurs in patients with poor coagulation function, it will be difficult to stop bleeding, and it will threaten the life safety of patients. Transjugular intrahepatic portal shunt (TIPS) can effectively reduce the portal vein pressure by establishing direct access between the portal vein and hepatic vein and is an effective means for the treatment of portal hypertension in cirrhosis.^4^

The guidelines indicate that clinicians should not routinely correct thrombocytopenia and coagulopathy before low-risk therapeutic paracentesis, thoracentesis, and routine upper endoscopy for variceal ligation in patients with hepatic synthetic dysfunction-induced coagulation abnormalities.^3,5^ As an effective means to reduce portal pressure, there are questions about TIPS’s safety for patients with poor coagulation clinically. This article will explore the safety and prognosis of TIPS in patients with mildly prolonged prothrombin time (PT).

## MATERIALS AND METHODS

### Study Design

The present observational study was conducted at Wuhan Union Hospital. The study protocol conforms to the 1975 Declaration of Helsinki and was approved by the Ethics Committee of Wuhan Union Hospital, Tongji Medical College, Huazhong University of Science and Technology. Waiver of informed consent was granted by the institutional review board of Wuhan Union Hospital, Tongji Medical College, Huazhong University of Science and Technology, because the data have been anonymized. We follow the Strengthening the Reporting of Observational Studies in Epidemiology (STROBE) guidelines for the reporting of observational studies.^6^

### Study Population and Data Collection

Patients who underwent TIPS treatment successfully with a confirmed diagnosis of portal hypertension were considered eligible for this study. Exclusion criteria included history of splenectomy or splenic embolism before TIPS, blood system diseases, portal vein thrombosis (PVT)—–Yerdel score ≥3, PT prolongation ≥6 seconds, or incomplete clinical data (preoperative PT was missing). Patients without exclusion criteria were enrolled.

In the diagnostic criteria of disseminated intravascular coagulation (DIC) formulated by the International Society on Thrombosis and Haemostasis (ISTH),^7^ 3 seconds and 6 seconds of PT prolongation are assigned different scores. Meanwhile, in the Child–Pugh grading standard, whether PT prolongation is greater than 6 seconds is also an important critical point.^8^ In addition, PT prolongation <3 seconds is generally considered to have no clinical significance (CS).^9^ Based on this, 253 patients with portal hypertension who received TIPS in Wuhan Union Hospital from November 2015 to May 2021 were retrospectively selected and divided into 2 groups according to the preoperative PT. The clinical data of 126 patients in the non-clinical significance (NCS) group (PT prolongation<3 seconds) and 127 patients in the CS (group (3 seconds ≤PT prolongation <6 seconds) were collected. The reference value of PT was defined as 11-13 seconds, and so the NCS group was defined as having 13 seconds <PT <16 seconds and the CS group was defined as having 16 seconds ≤ PT <19 seconds. In addition, patients with preoperative platelets no higher than 150 × 10^9^/L in the CS group were divided into severe thrombocytopenia group [platelet count (PLT) ≤50 × 10^9^/L)] and mild thrombocytopenia group (50 × 10^9^/L < PLT ≤150 × 10^9^/L) for subgroup analysis.

### Transjugular Intrahepatic Portosystemic Shunt Procedure

Transjugular intrahepatic portal shunt is performed by the same team of experienced physicians. The right internal jugular vein was punctured with rosch-uchida transjugular liver access set (RUPS)-100 (Cook Inc.; Bloomington, USA) puncture device, intubated to the hepatic vein through the vena cava, and the portal vein was punctured under fluoroscopy to establish the direct channel between the hepatic vein and the portal vein. Then, portasystemic shunt was established by balloon expansion (6-8 mm) and the stents were placed. All patients in this study were treated with membrane-covered stents to maintain long-term patency of stents.^5^ Placing a bare stent (Bard E-LUMINEXX Vascular Stent, Karlsruhe, Germany) followed by placing a coated stent (Fluency; Bard Inc., USA or Viabahn; Gore, USA). Portal vein pressure gradient (PPG) was measured before and after the shunt was established.

### Clinical Data of Observation

The clinical data of both groups were extracted from an electronic medical record system, including gender, age, cause of liver cirrhosis, Child–Pugh and model for end-stage liver disease (MELD) score, basic disease history, TIPS indications, history of endoscopic treatment, imaging examination (inner diameter of portal vein, inner diameter of splenic vein, long diameter of spleen, and PVT along with spleen, stomach, and kidney shunt), whether stomach esophagus varicose vein was embolized, preoperative postoperative PPG, and biochemical examination [total bilirubin (TBil), albumin (ALB), alanine aminotransferase, aspartate aminotransferase, serum creatinine, blood urea nitrogen, sodium ion concentration, PT, international normalized ratio (INR), PLT, and hemoglobin (Hb)].

### Statistical Analysis

Statistical Package for Social Sciences 26.0 software (IBM Corp.; Armonk, NY, USA) and R (version 4.0.3) statistical software were used for statistical analysis. The measurement data were expressed by mean ± SD (*X* ± *S*) to analyze whether all indicators were in line with normal distribution. If so, the *t*-test was used for differences between groups. If the distribution was not normal, the difference between groups was tested by the Mann–Whitney *U*-test. Statistical data were expressed in terms of number of cases and percentage [n (%)], and comparison between groups was performed by the *χ*^2^ or Fisher’s exact tests. Survival time was assessed using Kaplan–Meier (KM) curves with stent dysfunction as the primary endpoint and death, hepatic encephalopathy, and rebleeding or ascites as secondary endpoints. Bilateral *α* values less than 0.05 were considered statistically significant.

## RESULTS

### Baseline Characteristics

**Figure 1.**
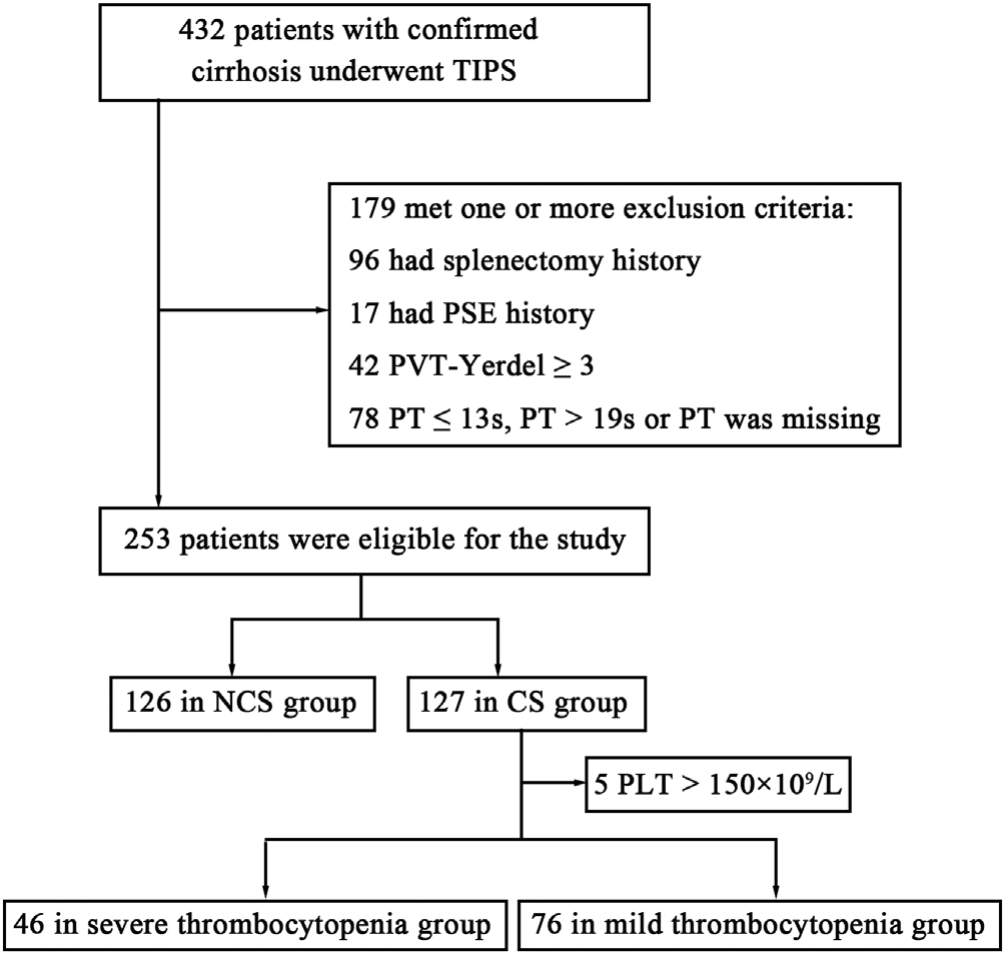
Flowchart of the patient selection protocol. CS, clinical significance; NCS, non-clinical significance; PLT, platelet count; PT, prothrombin time; PVT, portal vein thrombosis; TIPS, transjugular intrahepatic portal shunt.

Four hundred thirty-two consecutive patients with confirmed cirrhosis who received TIPS in our center were analyzed. After excluding 179 patients fulfilling 1 or more exclusion criteria, 253 patients were enrolled in the final cohort (Figure 1). In the 253 patients included, 66.0% were male, 48.2% were cirrhotic due to hepatitis B, and 81.4% were treated with TIPS for gastrointestinal bleeding. Age, Child–Pugh score, MELD score, history of endoscopic treatment, inner diameter of splenic vein, TBil, ALB, PT, INR, Hb, and PLT of patients in the 2 groups were statistically significant. The mean age of the NCS group was higher than that in the CS group (57.6 ± 11.7 vs. 54.5 ± 12.9, *P* = .045). The mean Child–Pugh score of the NCS group was lower than that in the CS group (6.9 ± 1.4 vs. 7.8 ± 1.3, *P* < .001). The MELD score in the NCS group was lower than that in the CS group (10.3 ± 3.3 vs. 12.4 ± 2.5, *P* < .001). The proportion of patients receiving endoscopy in the NCS group was higher than that in the CS group (18.3% vs. 7.9%, *P* = .014), as shown in Table 1. The mean inner diameter of the splenic vein in the NCS group was smaller than that in the CS group (11.0 ± 3.4 vs. 12.0 ± 3.7, *P* = .037). The average TBil in the NCS group was lower than that in the CS group (22.4 ± 16.2 vs. 29.5 ± 30.6, *P* = .021). The mean ALB of the NCS group was higher than that in the CS group (33.1 ± 4.9 vs. 29.9 ± 5.4, *P* < .001). The mean PT of the NCS group was lower than that of the CS group (14.9 ± 0.7 vs. 17.2 ± 0.8, *P* < .001). The mean INR of the NCS group was lower than that in the CS group (1.2 ± 0.1 vs. 1.4 ± 0.1, *P* < .001). The mean PLT of the NCS group was higher than that in the CS group (87.1 ± 48.3 vs. 67.8 ± 36.8, *P* < .001), as shown in Table 2.

### Complications and the Distribution of Prothrombin Time and INR

**Figure 2.**
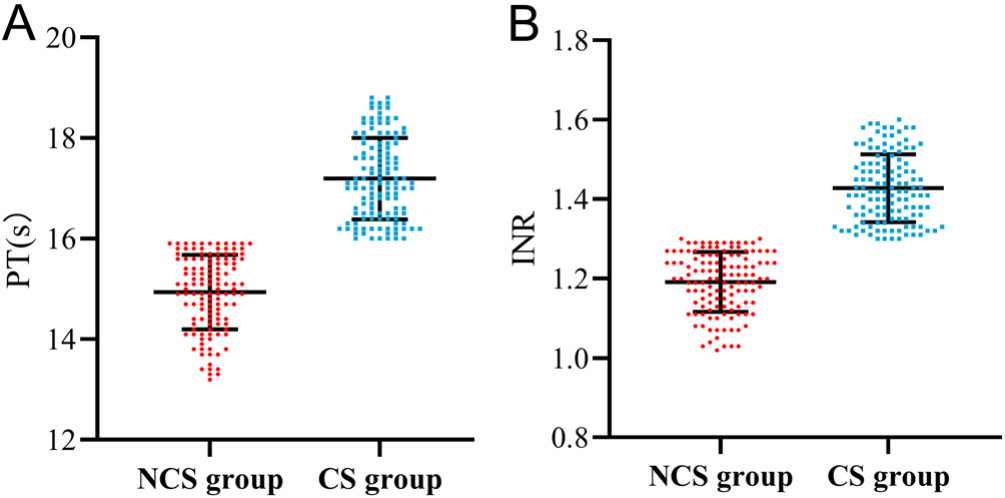
Scatter diagram of PT and INR distribution in patients. CS, clinical significance; NCS, non-clinical significance; PT, prothrombin time.

Transjugular intrahepatic portal shunt was successfully performed in all patients, with a technical success rate of 100%. After TIPS, mean PPG decreased significantly from 28.7 ± 5.9 mmHg to 12.5 ± 4.3 mmHg in the NCS group and from 27.4 ± 6.0 mmHg to 12.1 ± 4.7 mmHg in the CS group. There was no significant difference in the PPG decrease rate between the 2 groups (55.9 ± 12.4% vs. 56.7 ± 11.4%, *P* = .670). No serious complications such as abdominal bleeding, biliary tract bleeding, biliary peritonitis, or death occurred in other patients, irrespective of whether the patients were in the NCS group or the CS group. The distribution of PT and INR in the 2 groups is shown in Figure 2. The PT of the NCS group was 13.2 seconds and 15.9 seconds, respectively. The lowest PT in CS group was 16 seconds and the highest PT was 18.8 seconds. The lowest INR was 1.02 and the highest INR was 1.3 in the NCS group and the lowest INR was 1.3 and the highest INR was 1.6 in the CS group.

### Changes of Liver and Kidney Function After Operation

**Figure 3.**
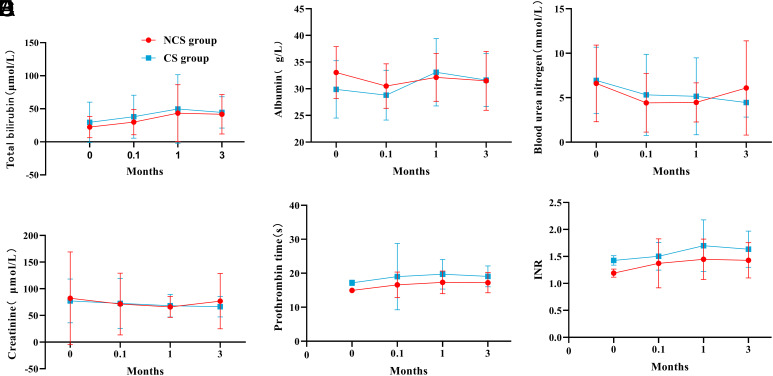
Comparison of liver and kidney functions between the 2 groups. (A) Total bilirubin; (B) albumin; (C) urea nitrogen; (D) creatinine; (E) prothrombin time; and (F) INR.

The TBil level in both groups continued to increase from preoperative to postoperative 1 month and began to decrease 1 month after surgery. Albumin decreased 1 week postoperatively but returned to preoperative levels quickly. Serum creatinine remained relatively stable in the 2 groups, while urea nitrogen decreased slightly after surgery. Prothrombin time and INR were slightly elevated after surgery, as shown in Figure 3.

### Survival Analysis

**Figure 4.**
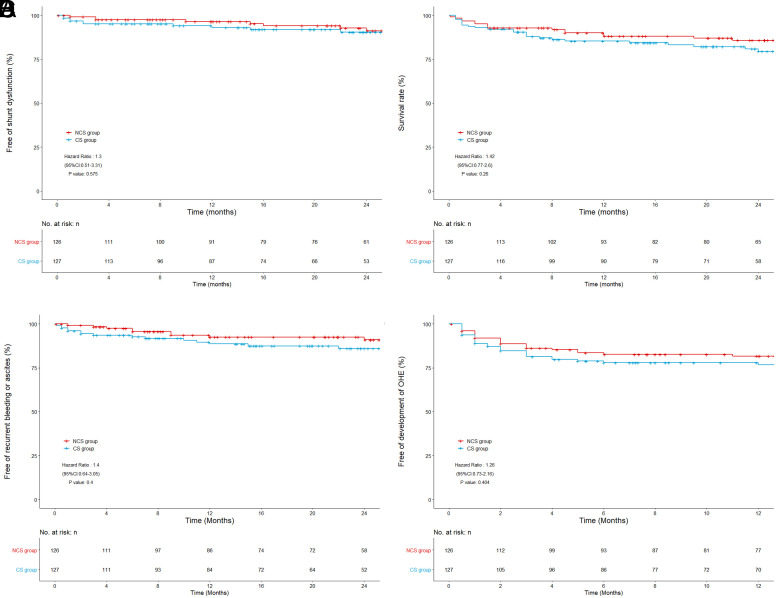
Kaplan–Meier curves of each endpoint. (A) Free of stent dysfunction; (B) survival rate; (C) free of recurrent bleeding; and (D) free of the development of overt hepatic encephalopathy (OHE).

The prognostic endpoints of the 2 groups were compared, including stent dysfunction, death, rebleeding, and hepatic encephalopathy, and KM curve analysis was performed, as shown in Figure 4. The KM curve showed no significant difference in prognostic endpoints between the 2 groups. The median follow-up time was 23.4 months [interquartile range (IQR) 10.0-33.3] in the NCS group and 20.5 months (IQR 8.1-34.6) in the CS group when stent dysfunction is the endpoint. Stent dysfunction occurred in 18 patients (7.1%) in the study cohort, including 8 patients (6.3%) in the NCS group and 10 patients (7.9%) in the CS group. Only 1 of 18 patients (CS group) had a new stent, while the remaining 17 underwent balloon dilatation stent plasty. The 1-year stent patency rates in the NCS and CS groups were 96.5% and 93.1%, respectively, and the 2-year stent patency rates were 91.2% and 90.4%, respectively. There was no significant difference in stent patency between the NCS and CS groups [hazard ratio (HR) = 0.77; 95% CI = 0.30-1.93; log-rank *P* = .575]. During follow-up, 43 patients (17.0%) died, including 18 (14.3%) in the NCS group and 25 (19.7%) in the CS group. The 1-year survival rates in the NCS and CS groups were 88.2% and 85.4% and the 2-year survival rates were 85.8% and 79.5%, respectively. There was no significant difference in survival between the NCS group and the CS group (HR = 0.71; 95% CI = 0.39-1.28; log-rank *P* = .255). Rebleeding occurred in 26 patients (10.3%), including 11 (8.7%) in the NCS group and 15 (11.8%) in the CS group. The rates of no rebleeding at 1 year in the NCS and CS groups were 92.5% and 88.6%, and no rebleeding rates at 2 years were 90.9% and 86.0%, respectively. There was no significant difference in the proportion of patients without rebleeding between the NCS and CS groups (HR = 0.71; 95% CI = 0.33-1.54; log-rank *P* = .392). During follow-up, 53 patients (20.9%) developed hepatic encephalopathy, including 29 (23.0%) in the NCS group and 24 (18.9%) in the CS group. The incidence of hepatic encephalopathy in the NCS and CS groups was 81.6% and 77.9% at 1 year, and 2-year rates were 79.2% and 76.8%, respectively. The difference was not statistically significant (HR = 0.80; 95% CI = 0.47-1.37; log-rank *P* = .404).

### Subgroup Analysis of the Clinical Significance Group

There are 122 patients whose platelets were not higher than 150 × 10^9^/L before operation in the CS group, including 46 patients in the severe thrombocytopenia group and 76 patients in the mild thrombocytopenia group. The mean preoperative platelet count was (38.04 ± 9.28) × 10^9^/L in the severe group and (77.33 ± 20.95) × 10^9^/L in the mild group, and the difference was statistically significant (*P* < .001). There was no significant difference in PT and INR between the 2 subgroups. The operative success rate of the 2 subgroups was 100%, and no severe complications such as abdominal bleeding, biliary tract bleeding, biliary peritonitis, or death occurred. In addition, there was no significant difference in prognosis between the 2 subgroups, as shown in Table 3.

### DISCUSSION

Existing guidelines indicate that coagulopathy need not be corrected before performing a low-risk puncture.^3^ It has been reported that ultrasound-guided pleural puncture is safe for patients with coagulopathy.^10^ However, the safety of TIPS for patients with coagulation dysfunction is worth exploring due to its high difficulty, long time, and complicated procedure. In this study, TIPS was found to be safe for patients with PT prolongation <6 seconds. We chose PT rather than INR to evaluate the coagulation function of patients because INR had some limitations in the application of liver disease.^11^ The international sensitivity index developed based on vitamin K antagonist (VKA) treatment samples might be significantly different from that developed based on liver disease samples.^12^

In our real-world study, TIPS was performed with a 100% success rate, and no patients developed puncture-related complications such as abdominal bleeding, bile duct injury, or biliary peritonitis. The reasons of no puncture-related complications are as follows:

1. Experienced surgeons can reduce the number of hepatic venipuncture, shorten the duration of surgery, reduce the damage to the patient’s body, and minimize the occurrence of complications.
2. Prothrombin time prolongation >3 seconds has CS. Among the included cases, the NCS group had no CS, while the CS group had CS. In addition, the average PT in the CS group was 17.2 ± 0.8 seconds without severe prolongation, and the coagulation function was not seriously abnormal.
3. Repeated gastrointestinal bleeding can cause large consumption of coagulation factors, resulting in transient prolongation of PT, while the synthesis of coagulation factors in the liver may be normal.
4. Prothrombin time reflects the function of the exogenous coagulation system but cannot completely reflect the overall function of the coagulation system. Prolongation of PT does not mean that the patient’s coagulation is impaired.
5. The use of anticoagulants such as heparin and coumarin can also cause PT prolongation.
6. Preoperative thrombocytopenia was moderate in both groups.^13^ Even in the CS group, the average thrombocytopenia was (67.8 ± 36.8) × 10^9^/L, which was at a relatively safe level. In addition, the INR in both groups was at a relatively safe level, and it was only 1.4 ± 0.1 in the CS group. In our real-world case, it is safe for patients with PT prolongation of 6 seconds to undergo TIPS, and no puncture-related complications have occurred. There is no need to correct the patient’s clotting function before TIPS in order to avoid delay of therapy.

Subgroup analysis of preoperative thrombocytopenia patients in the CS group showed that no puncture-related complications occurred in the 2 subgroups, and there was no significant difference in prognosis between the 2 subgroups. Two patients in the severe thrombocytopenia group had preoperative platelets lower than 20 × 10^9^/L (11 × 10^9^/L and 18 × 10^9^/L, respectively), which were at risk of spontaneous bleeding.^14^ However, PT of the 2 patients was 17.4 seconds and 17 seconds, respectively, and the INR of the 2 patients was 1.45 and 1.41, respectively, which was in the relatively safe range. After a comprehensive analysis of the coagulation function of the patient, we decided to perform TIPS on the patient, and the fact proved that there were no complications.

In addition, there was no significant difference in postoperative function of liver and kidney between the 2 groups. The liver damage caused by TIPS puncture was slight. One week after surgery, TBil slightly increased and ALB slightly decreased, but as time went on, liver function gradually returned to the preoperative level. Creatinine and urea nitrogen decreased slightly, indicating that the patient’s renal function improved after surgery. Trends in liver and kidney function further demonstrated the safety of TIPS in both groups.

The KM curve was used to analyze the prognosis of patients. No significant difference was found in the prognosis of stent dysfunction, rebleeding, death, and hepatic encephalopathy between the 2 groups. In a study involving 495 patients, the 1-year stent patency rate was 93%.^15^ In our study, the 1-year stent patency rate of the NCS group and CS group was 96.5% and 93.1%, respectively, indicating that the stent patency rate was at a high level. Studies have shown that the rate of rebleeding after TIPS was 10%-20%,^16^ and a total of 26 patients (10.2%) in this study experienced rebleeding during the follow-up period. The 1-year mortality rate of the NCS group and the CS group was 14.2% and 20.5%, respectively, and the 1-year incidence of hepatic encephalopathy was 19.4% and 22.1%, respectively, both within the range reported by most studies.^17-19^ This further suggests that although there was no significant difference in the prognosis between the 2 groups, both groups had a good prognosis.

The limitations of this study are as follows. This study is a single-center retrospective study, with inevitable selection bias, and only bare stents combined with coated stents were used instead of Viatorr stents, because Viatorr stents were not available in China at that time. However, some researchers have confirmed that dual stents can be used as an alternative when the availability of Viatorr stents is limited.^20,21^

## CONCLUSION

Transjugular intrahepatic portal shunt is safe for portal hypertension patients with PT prolongation <6 seconds. There was no significant difference in prognosis between the NCS group and the CS group.

## Figures and Tables

**Table 1.** Comparison of Demographic and Clinical Characteristics of the 2 Groups

Variables	All Patients (n = 253)	NCS Group (n = 126)	CS Group (n = 127)	*P*
Gender				.101
Male [*n* (%)]	167	77	90	
Female [*n* (%)]	86	49	37	
Age (years)	56.0 ± 12.4	57.6 ± 11.7	54.5 ± 12.9	.045
Child–Pugh score	7.3 ± 1.4	6.9 ± 1.4	7.8 ± 1.3	<.001
MELD score	11.3 ± 3.1	10.3 ± 3.3	12.4 ± 2.5	<.001
Comorbid diseases				.930
Hypertension [*n* (%)]	32	16	16	
Diabetes [*n* (%)]	54	29	25	
Malignant tumor [*n* (%)]	22	12	10	
History of endoscopic treatment [*n* (%)]	33	23	10	.014

CS, clinical significance; NCS, non-clinical significance.

**Table 2.** Biochemical Characteristics Between the 2 Groups

Variables	All Patients (n = 253)	NCS Group (n = 126)	CS Group (n = 127)	*P*
CT—inner diameter of the portal vein (mm)	15.2 ± 3.4	14.9 ± 3.0	15.6 ± 3.8	.094
CT—inner diameter of the splenic vein (mm)	11.5 ± 3.5	11 ± 3.4	12 ± 3.7	.037
CT—long diameter of the spleen (cm)	16.0 ± 3.2	15.7 ± 3.0	16.4 ± 3.3	.176
Spleen, stomach, and kidney shunt [*n* (%)]	52(20.6)	24 (19.0)	28 (22.0)	.555
Portal vein thrombosis [*n* (%)]	73(28.9)	33 (26.2)	40 (31.5)	.352
Preoperative PPG (mmHg)	28.1 ± 6.0	28.7 ± 5.9	27.4 ± 6.0	.17
Postoperative PPG (mmHg)	12.3 ± 4.5	12.5 ± 4.3	12.1 ± 4.7	.552
TBil (μmol/L)	26.0 ± 24.7	22.4 ± 16.2	29.5 ± 30.6	.021
ALB (g/L)	31.5 ± 5.4	33.1 ± 4.9	29.9 ± 5.4	<.001
ALT (U/L)	38.6 ± 79.0	40.7 ± 107.9	36.5 ± 30.4	.667
AST (U/L)	48.7 ± 74.9	51.4 ± 98.2	46 ± 40.2	.573
CRE (μmol/L)	79.4 ± 63.9	84.5 ± 84.7	74.4 ± 31.3	.21
BUN (mmol/L)	6.8 ± 4.0	6.6 ± 4.3	6.9 ± 3.7	.51
Na^+^(mmol/L)	138.4 ± 4.5	138.7 ± 4.1	138 ± 4.9	.223
PT (seconds)	16.1 ± 1.4	14.9 ± .7	17.2 ± .8	<.001
INR	1.3 ± .1	1.2 ± .1	1.4 ± .1	<.001
Hb (g/L)	84.4 ± 26.3	87.8 ± 27.9	81 ± 24.1	.039
WBC (×10^9^/L)	4.0 ± 2.4	4.0 ± 2.1	4.0 ± 2.8	.959
PLT (×10^9^/L)	77.4 ± 43.9	87.1 ± 48.3	67.8 ± 36.8	<.001

ALB, albumin; ALT, alanine aminotransferase; AST, aspartate aminotransferase; BUN, blood urea nitrogen; CRE, serum creatinine; CS, clinical significance; CT, computerized tomography; Hb, hemoglobin; Na^+^, sodium ion concentration; NCS, non-clinical significance; PLT, platelet count; PPG, portal vein pressure gradient; PT, prothrombin time; TBil, total bilirubin; WBC, white blood cell.

**Table 3.** Subgroup Analysis of the CS Group

Variables	Severe Group	Mild Group	*P*
PLT (×10^9^/L)	38.04 ± 9.28	77.33 ± 20.95	<.001
PT (seconds)	17.37 ± 0.78	17.10 ± 0.81	.070
INR	1.44 ± 0.09	1.42 ± 0.09	.227
Complications	0	0	—
Survival analysis			
Shunt dysfunction			.567
Death			.729
Recurrent bleeding			.458
Development of OHE			.346

CS, clinical significance; PLT, platelet count; PT, prothrombin time.
